# ARA-net: an attention-aware retinal atrophy segmentation network coping with fundus images

**DOI:** 10.3389/fnins.2023.1174937

**Published:** 2023-04-27

**Authors:** Lei Chen, Yuying Zhou, Songyang Gao, Manyu Li, Hai Tan, Zhijiang Wan

**Affiliations:** ^1^Second College of Cinical Medicine, Nanchang University, Nanchang, Jiangxi, China; ^2^Information Engineering College, Nanchang University, Nanchang, Jiangxi, China; ^3^School of Computer Science, Nanjing Audit University, Nanjing, Jiangsu, China; ^4^Industrial Institute of Artificial Intelligence, Nanchang University, Nanchang, Jiangxi, China

**Keywords:** retinal atrophy, segmentation, self-attention, multi-scale, 2D fundus images

## Abstract

**Background:**

Accurately detecting and segmenting areas of retinal atrophy are paramount for early medical intervention in pathological myopia (PM). However, segmenting retinal atrophic areas based on a two-dimensional (2D) fundus image poses several challenges, such as blurred boundaries, irregular shapes, and size variation. To overcome these challenges, we have proposed an attention-aware retinal atrophy segmentation network (ARA-Net) to segment retinal atrophy areas from the 2D fundus image.

**Methods:**

In particular, the ARA-Net adopts a similar strategy as UNet to perform the area segmentation. Skip self-attention connection (SSA) block, comprising a shortcut and a parallel polarized self-attention (PPSA) block, has been proposed to deal with the challenges of blurred boundaries and irregular shapes of the retinal atrophic region. Further, we have proposed a multi-scale feature flow (MSFF) to challenge the size variation. We have added the flow between the SSA connection blocks, allowing for capturing considerable semantic information to detect retinal atrophy in various area sizes.

**Results:**

The proposed method has been validated on the Pathological Myopia (PALM) dataset. Experimental results demonstrate that our method yields a high dice coefficient (DICE) of 84.26%, Jaccard index (JAC) of 72.80%, and F1-score of 84.57%, which outperforms other methods significantly.

**Conclusion:**

Our results have demonstrated that ARA-Net is an effective and efficient approach for retinal atrophic area segmentation in PM.

## 1. Introduction

The eyes are one of the essential sensory organs in humans; many people worldwide have myopia, which causes many inconveniences in their lives. Holden et al. (2016) performed a meta-analysis of myopia prevalence. They predicted that by 2050, 49.8 and 9.8% of the world’s population would suffer from myopia and high myopia, respectively. High myopia has the risk of deteriorating into pathological myopia. Retinal changes caused by myopia include fundus tessellation, parapapillary atrophy, optic disc tilting, myopic maculopathy, and retinal detachment. Retinal atrophy is a condition that leads to the loss of retinal layers, affecting vision quality. It is associated with choroidal retinal thinning and attenuation of the parapapillary retinal pigment epithelium (RPE) adjacent to the optic nerve head (ONH). Myopia, glaucoma, and age-related macular degeneration (AMD) are among the diseases that can cause retinal atrophy (Jonas et al., 1988; Manjunath et al., 2010; Srinivas et al., 2018). Accurate segmentation of retinal atrophic regions from OCT or fundus images is essential for eye condition diagnosis, monitoring, and treatment. It enables personalized interventions and plays a crucial role in improving the overall management of these ocular conditions.

The degree of retinal atrophy is a valuable medical assessment indicator as it is correlated closely with the severity of ophthalmic diseases and conditions, including glaucomatous optic nerve damage, visual field defects, and myopia (Park et al., 1996; Uchida et al., 1998; Dai et al., 2013). Consequently, the segmentation of retinal atrophic regions has become a significant part of diagnosing ophthalmic diseases. Although experienced ophthalmologists can give accurate results, manual segmentation is laborious and time-consuming, and different ophthalmologists might make different treatments. The development of an automatic segmentation model to accurately segment the retinal atrophy regions is thus vital, as it offers a reliable, efficient, and arguably more consistent diagnosis for ophthalmic diseases. The automatic segmentation models always adopt fundus images to perform the segmentation task of retinal atrophy areas. Compare with three-dimensional (3D) fundus image, two-dimensional (2D) fundus image are more widely available and easier to be acquired. The 3D fundus images require special equipment and technology that may not be accessible or affordable for many clinics or researchers. In addition, the 2D fundus images can provide sufficient information for segmenting retinal atrophy areas, which are mainly located on the surface of the retina.

In previous studies, most segmentation models are based on traditional image segmentation algorithms with manually designed features. Lu et al. (2010) segmented and quantified the optic disc and parapapillary area automatically using a combination of techniques, such as scanning filter, thresholding, region growing, and a modified Chan-Vese model (Chan and Vese, 2001) with a shape constraint. Li et al. (2018) proposed a novel parapapillary atrophy segmentation algorithm that utilizes evenly-oriented radial line segments and ellipse fitting. Although the traditional methods utilize machine learning to implement the image segmentation algorithm, most of them require manual feature selection and are not end-to-end solutions. Recent strides in deep learning have enabled the utilization of deep learning-based techniques in the medical domain, surpassing traditional methods in image segmentation with a higher degree of accuracy. Current mainstream deep learning methods for object segmentation can be divided into convolution-based and transformer-based methods. Long et al. (2014) proposed full convolutional networks (FCN), a model foundation of many segmentation networks for pixel-wise semantic segmentation tasks. Transformer-based (Vaswani et al., 2017) image segmentation models have emerged because they learn a global understanding of images which facilitates image segmentation models to achieve accurate segmentation results.

Deep learning models, especially UNet, have been widely adopted in various studies for segmenting areas of retinal atrophy based on fundus images. The UNet is a convolutional neural network designed for biomedical applications (Ronneberger et al., 2015). The core module, the FCN, utilizes the skip connections between the encoder and decoder to improve model performance. Due to its low demand on dataset size and the U-shaped structure containing contextual information, UNet has become a prevalent choice for medical segmentation and yields promising results. Furthermore, variations of UNet have been proposed to enhance the model performance. Zhou et al. (2018) proposed UNet++ and re-designed the skip pathways to reduce the semantic gap between the feature maps of the encoder and decoder networks. Guo et al. (2020) proposed a novel Lesion-aware segmentation network inspired by the UNet encoder-decoder structure and contained a binary classifier. The feature flows were integrated into the decoder to absorb various scales of feature maps. Ruben et al. (2020) evaluated the detection of pathological myopia (PM) using deep learning and the semantic segmentation of myopia-related lesions from fundus images. They used UNet++ as their network and used ResNet-18 as encoders. Chai et al. (2020) proposed a novel multi-task fully convolutional network (MFCN) model for peripapillary atrophy area segmentation from retinal images by transforming the atrophic area into two regions with relatively regular and uniform shapes. Wan et al. (2021) proposed OT-Unet, combining parallel partial decoder, edge attention, and reverse attention modules to enhance the segmentation accuracy.

**Figure 1:**
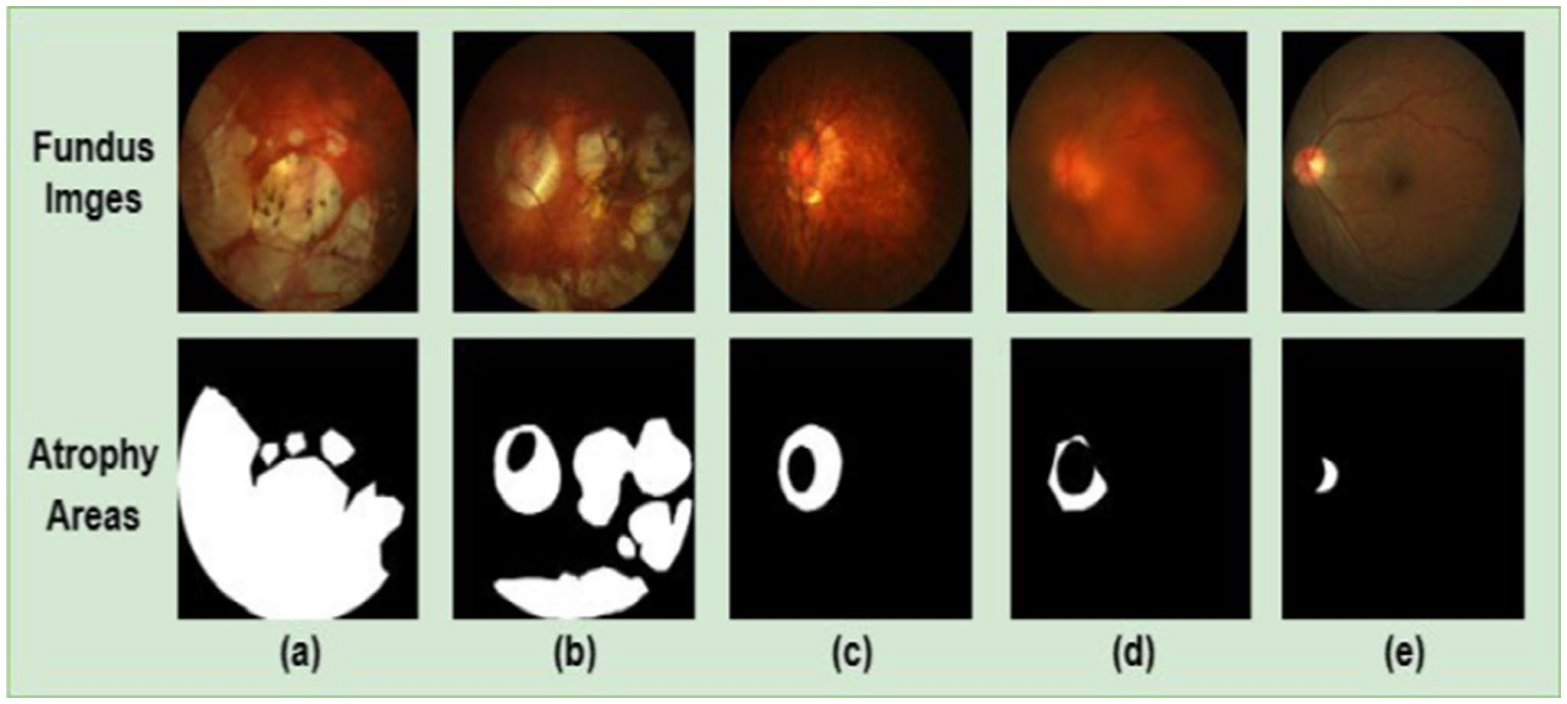
2D fundus images (top part) and the corresponding retinal atrophic areas (bottom part). Subfigures **(A–D)** are fundus images of patients with PM. Subfigure **(E)** is a fundus image of patients with non-pathological myopia. PM images have more significant retinal atrophic regions than non-pathological myopia images.

Although the existing UNet-based retinal area segmentation algorithms achieved good results, the performance of the segmentation model is challenged by the following characteristics of retinal atrophic regions in the 2D fundus image, such as blurred boundaries, irregular shape, and size variation. These characteristics can make the segmentation models challenging to segment the areas accurately. As depicted in Figure 1, the top and bottom parts show a 2D fundus image and the corresponding retinal atrophy areas (i.e., areas in white), respectively. The bottom parts of subfigures (a) and (b) exhibit larger areas of retinal atrophy, while the bottom parts of subfigures (c), (d), and (e) show significantly reduced areas of atrophy. From the figure, we know that retinal atrophic areas can vary significantly in size from patient to patient, and the areas of each patient are randomly distributed in the fundus image. In addition, the model’s generalization ability is restricted by the limited availability of annotated fundus image datasets.

To overcome these challenges, we proposed an attention-aware retinal atrophy segmentation network based on UNet structure, containing an encoder, a decoder, skip self-attention (SSA) connection blocks and multi-scale feature flow (MSFF), to segment retinal atrophic areas from 2D fundus image. The SSA connection block contains two distinct components: a shortcut and a parallel polarized self-attention (PPSA) block. The shortcut helps to preserve the original features, while the PPSA block can enhance feature learning capabilities. The PPSA block can capture contextual information over long distances and map semantic information from the encoder in both channel and spatial dimensions. Thus, it enables the model to learn features more robustly for alleviating the challenges, such as blurred boundaries and irregular shapes of the retinal atrophy region. Furthermore, a MSFF is added between the SSA connection blocks to address the challenge of size diversity in the retinal atrophy region. In addition, the transfer learning strategy and data augmentation are introduced to improve the performance and generalization ability of the model. Due to the significant time and computational resources required for developing effective deep learning models, transfer learning has become a widely used strategy. Transfer learning uses the knowledge gained from pre-trained models to improve the performance of new tasks. Pre-trained models can train models efficiently for new segmentation tasks, reducing time and computational costs. And the transfer learning can help combat overfitting by providing a starting point for the model and reducing the reliance on training data. To improve the sensitivity rate of the retinal atrophy segmentation, a customized hybrid loss was employed to assign a higher weight to false negative detections. It enabled the algorithm to be more sensitive to false negative detections, thus leading to more precise segmentation results. The main contributions of our work can be enumerated as follows:

1. We proposed a novel skip connection block named the SSA connection block, which can be easily integrated into existing UNet-based architectures. The SSA connection block can better capture the global structure of the retinal atrophy, allowing the model to learn features more robustly. It is capable of dealing with blurred boundaries and irregular shapes of the retinal atrophic region. Additionally, it only requires a minimal increase in computational overheads.
2. We proposed an MSFF between the SSA connection blocks, allowing the network to capture multi-scale semantic information and significantly enhancing the self-attention mechanism’s ability to capture multi-scale spatial and channel features. Thus, it improves the segmentation performance and more accurate detection of retinal atrophy and effectively addresses the challenge of size diversity in the retinal atrophic region.
3. We introduced a learning strategy to improve the performance and generalization of the model. By employing pre-trained models with large datasets to initialize the model weights, adaptation to new datasets with reduced training data is expedited. This improves segmentation accuracy, shortens training time, and reduces computing resources.

## 2. Materials and methods

### 2.1. Data preparation

Retinal images were obtained from the “Detection of Pathological Myopia from Retinal Images” challenge (iChallenge-PALM) held at the IEEE International Symposium on Biomedical Imaging, organized in 2019 (Fu et al., 2019). The training and validation datasets contain 311 fundus images and 271 fundus images, respectively. The Zeiss VISUCAM device took these fundus images at an angle of 45° with a resolution of 2,124 × 2,056, or 30 ° angle with a resolution of 1,444 × 1,444. To improve the computational efficiency and conserve computing resources, all fundus images were resized to 512 × 512 and normalized to facilitate faster and more stable processing by the neural network. Finally, a logical AND operation of the network’s predicted mask and the original image was performed to generate the resulting color output.

### 2.2. Model architecture

**Figure 2:**
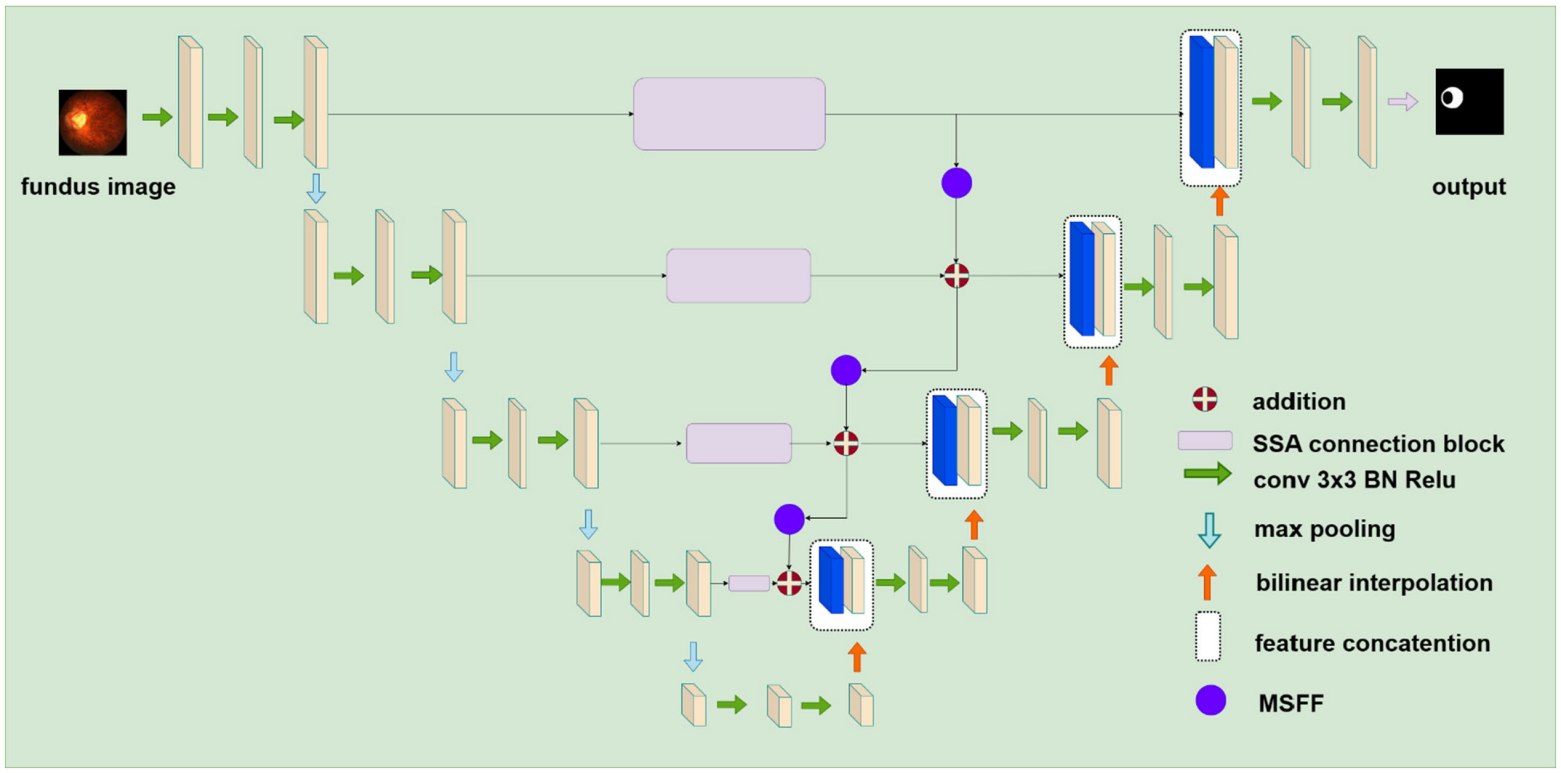
The structure of the proposed deep learning network for segmenting retinal atrophic areas from 2D fundus images. The encoder on the left extracts features, and the decoder on the right recovers image details. The SSA connection block represented by purple rectangular flows the feature from the encoder to the decoder.

Figure 2 illustrates the proposed deep learning network for segmenting retinal atrophic areas from 2D fundus images. The proposed network consists of an encoder, a decoder, and the PPSA block. The encoder is responsible for extracting the features of an input image, while the decoder is responsible for recovering the image details and capturing the boundaries of the retinal atrophy region. The PPSA blocks act as a bridge between the encoder and decoder, providing a source of feature information to the decoder. This connection is essential in allowing the network to reconstruct high-frequency details.

#### 2.2.1. UNet backbone

UNet is a fully convolutional network architecture for medical research applications such as segmentation and classification. It consists of an encoder and a decoder based on convolutional neural networks (CNNs). The encoder utilizes 3 × 3 convolutional layers, batch normalization, 2 × 2 max-pooling layers, and ReLu activation functions to extract features from the input image. In contrast, the decoder contains transposed convolutional layers (bilinear interpolation), 3 × 3 convolutional layers, batch normalization, 2 × 2 max-pooling layers, and ReLu activation functions to reduce the number of channels and to segment pixels into different regions. The skip connections between the encoder and decoder networks further facilitate the flow of low-level features from the encoder to the decoder and improve segmentation performance. UNet can learn complex feature representations and provide high-quality performance in biomedical image segmentation tasks.

#### 2.2.2. SSA connection block

In the original UNet architecture, features from the encoder flow directly to the decoder *via* a skip connection. However, the features received by the decoder are mostly background information, do not provide meaningful semantic information, and are not on the same semantic level as the encoder. To overcome this problem and make the network more attentive to atrophic region edges and shape information, we propose the SSA Connection block, which can better capture long-range dependencies in the feature maps. In addition, the parallel polarization design of the self-attention mechanism allows the block to learn feature maps in both the spatial and channel dimensions, allowing it to capture features of the retinal atrophy region effectively.

**Figure 3:**
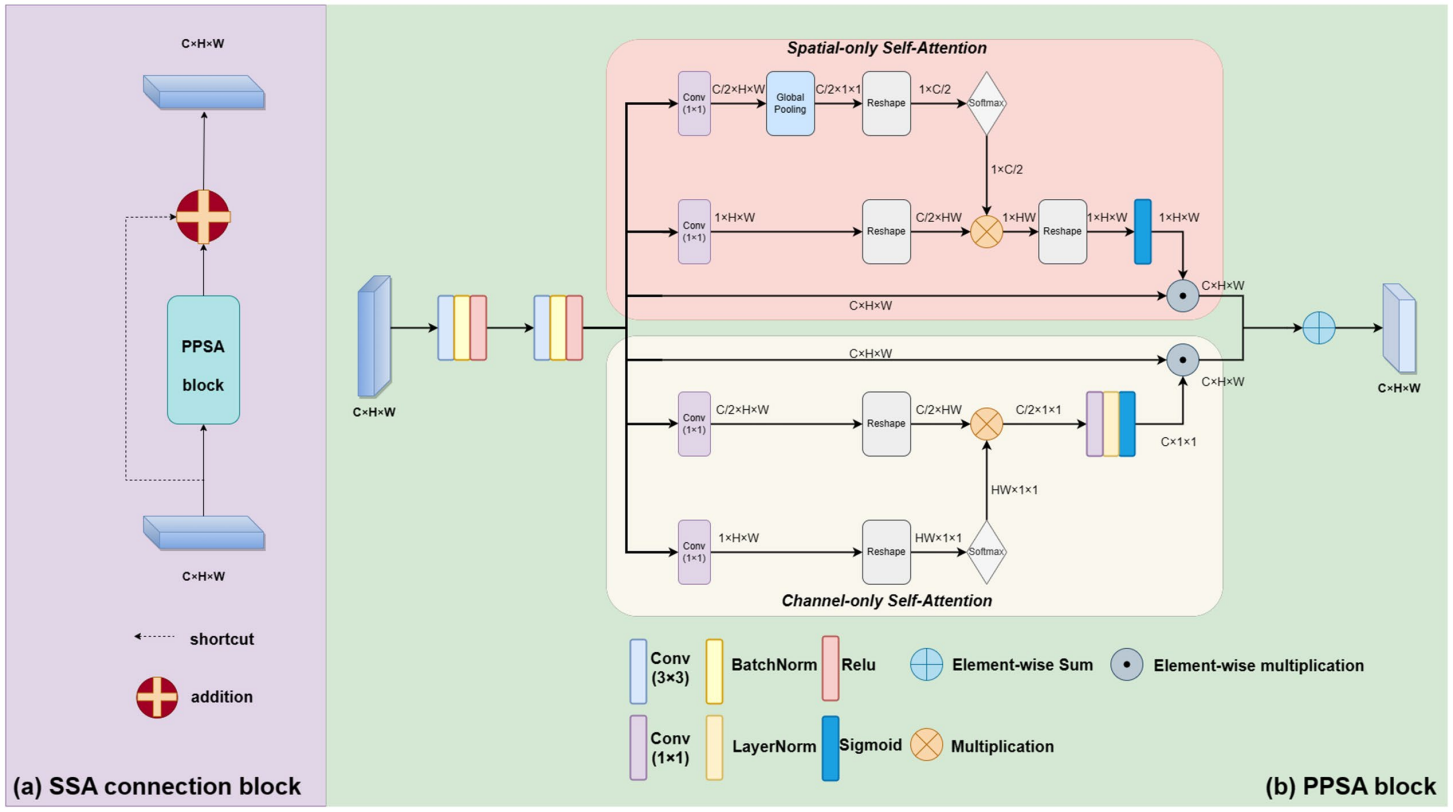
The proposed novel SSA connection block. As shown in subfigure **(A)**, it contains a shortcut path and PPSA block paths. The PPSA block is shown in subfigure **(B)**.

Our skip self attention blocks not only retain the original Unet skip connection which allow direct connections between the encoder and decoder layers, preserving low-level features that can then be combined with high-level features, but also include a PPSA branch in which contains two key modules: polarized self-attention and mapping enhancement. Figure 3 shows the proposed SSA block, subfigure (a) gives a shortcut path and PPSA block paths, and subfigure (b) introduces the detailed structure of the PPSA block. As shown in subfigure (b), the PPSA block has two convolution layers, followed by polarized self-attention, which contains two branches: Spatial-only self-attention and Channel-only self-attention. Polarized filtering is a design technique in deep learning that involves maintaining the internal resolution of both the channel and spatial attention computations, while reducing the dimensionality of the input data. This helps to filter out irrelevant data and preserve important details, allowing the model to focus on the most important features. Mapping enhancement is a design strategy that involves mapping the output of the model to a distribution that more closely resembles a typical fine-grained regression. In retinal atrophy segmentation, the output can be mapped to a 2D Binomial distribution that represents the probability of each pixel belonging to the segmented object. This design helps the model to better fit the output to the desired distribution, resulting in more accurate predictions. The computation method of the spatial-only self-attention is given as follows:


(1) $$\begin{array}{l} Out_{sp}\left(X\right)=Sigmoid\\ \left({\left(Softmax{\left(GP\left(Conv_{1\times 1}\left(X\right)\right)\right)}^{R}\times {\left(Conv_{1\times 1}\left(X\right)\right)}^{R}\right)}^{R}\right), \end{array}$$

where $Conv_{1\times 1}$ is standard convolutional layer using 1×1 convolution, Softmax and Sigmoid are activation functions, R is tensor reshape operation, and GP is global average operation. The computation method of the channel-only self-attention is described as follows:


(2) $$\begin{array}{l} Out_{ch}\left(X\right)=Sigmoid\\ \left(LN\left(Conv_{1*1}{\left(Softmax{\left(Conv_{1*1}\left(X\right)\right)}^{R}\times Conv_{1*1}\left(X\right)\right)}^{R}\right)\right) \end{array}$$

where LN means layer normalization. The final output of the PPSA block is listed as follows:


(3) $$PPSA\left(X\right)=CBR\left(CBR\left(Out_{sp}\left(X\right)\underset{sp}{⊙}X+Out_{ch}\left(X\right)\underset{ch}{⊙}X\right)\right),$$

where CBR is a combination of the convolution layer, BatchNorm, and ReLu activation function, $\underset{sp}{⊙}$ and $\underset{ch}{⊙}$ are multiplication operators in spital and channel dimensions, respectively. The output of the skip connection block is given as follows:


(4) $$\begin{array}{r} Block_{skip}\left(X\right)=X+PPSA\left(X\right). \end{array}$$

#### 2.2.3. Multi-scale feature flow

The MSFF is a critical component of our proposed model, as it integrates features from multiple resolutions to understand the input data comprehensively. It enhances the ability of the self-attention mechanism to capture spatial and channel features at different resolutions. This is especially important in retinal atrophy segmentation, where the size and shape of the atrophic lesions can vary greatly. MSFF is implemented using a 1 × 1 convolutional layer followed by a 2 × 2 average pooling layer that downsamples the high-resolution feature map to a low-resolution feature map. This affects the resolution of the feature maps, but by inserting MSFF blocks between SSA blocks to enhance the model’s ability to perceive multi-scale semantic information. In this case, the MSFF blocks reduce the resolution of the feature maps without affecting the overall perceptual capability of the network, as the feature maps from different scales are already fused together. This ultimately improves the model’s segmentation accuracy, thus mitigating information loss and distortion. The output of the skip connection block is given as follows:


(5) $$MSFF\left(X\right)={\mathrm{AP}}_{2*2}\left(Conv_{1*1}\left(X\right)\right).$$

### 2.3. Loss function

The image segmentation task can be viewed as a pixel-level classification problem, which refers to the process of categorizing every pixel in an image into its corresponding semantic class, such as atrophic regions or healthy tissue. In the context of retinal atrophy segmentation, pixel-level prediction enables the identification and localization of atrophic regions with high precision. This is achieved by assigning every pixel in the image a label based on its semantic class (i.e., atrophic or healthy tissue), allowing for the creation of a segmented image highlighting the area of atrophic regions. Therefore, we used binary cross-entropy loss (BCE), shown in Eq. 6, as a part of the loss function. However, since the pixel number of the region of interest (ROI) generally accounts for less than 20% of the total pixel number in an image, only using the BCE loss function alone may not be sufficient for accurate ROI classification as it prioritizes overall accuracy over foreground or background classification. In addition, the foreground pixels in the most fundus images, which includes the retina and blood vessels, makes up about 90% of the image. The pixels of atrophic area, makes up less than 10% of the image. The problem with this sample imbalance is that if a model is trained using a simple cross-entropy loss function, which is a common loss function used in image classification tasks, the model may learn to predict the outcome tendency as the foreground. In other words, the model may learn to ignore the atrophic area and only focus on the larger, more prominent foreground. This might be a problem in medical image analysis, because it may lead to false negatives or missed diagnosis. The Tversky Loss introduces the Tversky Index, with two adjustable parameters, α and β, that balance false positives and false negatives while prioritizing false negatives for small ROIs. Incorporating the Tversky Loss into the loss function can improve the accuracy of ROI classification, leading to better overall performance in the task. Therefore, we introduce the Tversky Loss ( $L_{TL}$ ) (Abraham and Khan, 2018), shown in Eq. 7, in the loss function to improve sensitivity for small ROIs. The Tversky Loss introduces the Tversky Index, with two adjustable parameters, α and β, that balance false positives and false negatives while prioritizing false negatives for small ROIs. The final combination loss function is shown in Eq. 8,


(6) $$L_{BCE}=-\frac{1}{N}\overset{N}{\underset{i=1}{\sum }}\left[\left(1-y_{i}\right)\cdot \log\left(1-\left(p\left(y_{i}\right)\right)\right)+y_{i}\cdot \log\left(p\left(y_{i}\right)\right)\right],$$

(7) $$\begin{array}{l} L_{TL}=1-\\ \frac{\sum_{i=1}^{N}p_{a}\left(y_{i}\right)g_{a}\left(y_{i}\right)+\xi }{\sum_{i=1}^{N}p_{a}\left(y_{i}\right)g_{a}\left(y_{i}\right)+\alpha \sum_{i=1}^{N}p_{b}\left(y_{i}\right)g_{a}\left(y_{i}\right)+\beta \sum_{i=1}^{N}p_{a}\left(y_{i}\right)g_{b}\left(y_{i}\right)+\xi }, \end{array}$$

(8) $$L_{f}=\mu \times L_{BCE}+\left(1-\mu \right)\times L_{TL},$$

where the total number of pixels is represented by *N*, and the pixels *y_i_* are labeled by 1 to represent the pixels located in the atrophic area. The pixels in the image background are labeled by 0. *p_a_*(*y_i_*) is the probability of the pixel belonging to the atrophic area, and *p_b_*(*y_i_*) is used to denote the probability of the pixel in the image background. *g_a_*(*y_i_*) is the probability of the pixel in ground truth belonging to the atrophied area, and *g_b_*(*y_i_*) is the probability of the pixel in ground truth belonging to the background. We set μ to 0.75, α to 0.6 and β to 0.4.

## 3. Experiments and results

### 3.1. Evaluation metric

To evaluate the performance of our model and compare it with other models, we use six evaluation metrics: Dice coefficient (DICE) (Dice, 1945), Jaccard index (JAC, IoU) (Jaccard, 1912), Precision (PRE), Sensitivity (SEN, also known as recall), Accuracy (ACC) and F1-score. The six metrics are defined as follows:


(9) $$DICE=\frac{2\cdot |G\cap P|}{|G|+|P|}=\frac{2\cdot TP}{FP+FN+2\cdot TP},$$

(10) $$JAC=\frac{\left|G\cap P\right|}{\left|G\cup P\right|}=\frac{TP}{FP+FN+TP},$$

(11) $$ACC=\frac{TP+TN}{TP+TN+FP+FN},$$

(12) $$PRE=\frac{TP}{TP+FP},$$

(13) $$SEN=\frac{TP}{TP+FN},$$

(14) $$F1-score=\frac{2\cdot PRE\cdot SEN}{PRE+SEN},$$

where G and P refer to ground truth and predicted mask, respectively, TP, TN, FP, and FN represent the number of true positives, true negatives, false positives, and false negatives, respectively. The DICE and JAC metrics are used to measure how accurate the predicted segmentation produced by a model is when compared to the ground truth. The scores range from 0 to 1, with higher scores indicating a higher level of accuracy. These metrics are useful for evaluating model performance and comparing different models against each other. The F1-score is a combination of PRE and SEN. PRE measures how often the model accurately identifies positives, while SEN measures how often the model identifies true positives and is a measure of sensitivity. The F1-score ranges from 0 to 1, with higher scores indicating a better balance between precision and sensitivity. This metric is important because it provides an overall assessment of how well the model is able to identify true positives and true negatives while minimizing false positives and false negatives. Overall, these metrics are important for evaluating a model’s ability to accurately identify the target object without including false positives or false negatives.

### 3.2. Implementation details

The implementation of the proposed network is based on the Pytorch computing library and was performed on a system equipped with RTX A5000, offering 24 GB of memory, and Tesla P100 with 16 GB of memory. To improve the generalization performance and prevent overfitting of our model, we employed various data augmentation techniques, including random flipping and image cropping. By setting a horizontal and vertical flip probability of 0.5, we randomly flip each image, while cropping the training images to 320 × 320 allows us to extract multiple sub-images from a single image, increasing the quantity and variability of our training data. To optimize the network, the initial learning rate of the AdamW optimizer was set to 0.008, the betas to (0.9, 0.999) and the weights decayed to 1e-4. Mini-batch is a popular optimization algorithm used for the training of deep neural networks. It works by dividing the training data into smaller batches and updating the model parameters based on the gradients computed from each batch. The batch size in our experiment was set to 32, ensuring comparable training and consistency from all experiments.

### 3.3. Ablation study

#### 3.3.1. Effectiveness of blocks

We conducted an ablation study to investigate the contribution of each block to the model performance. We began with the native UNet and then sequentially added novel SSA connection blocks and MSFF to the architecture to show the effectiveness of each of our proposed blocks. For the experiments conducted, four groups of models were developed, each consisting of an ARA-Net model combined with a different backbone ResNet18 (ARA-Net(ResNet18)), MobileNet-v3 (ARA-Net(MobileNet-v3)), and EfficientNet-b3 (ARA-Net(EfficientNet-b3)). ResNet18, with its 18 layers and residual connections, allows the network to learn deeper features and prevent vanishing gradients. MobileNet-v3, on the other hand, uses depth wise separable convolutions to reduce the number of parameters and computation needed while maintaining good accuracy. EfficientNet-b3 uses a compound scaling method to optimize the network architecture for different resource constraints, making it a relatively lightweight choice for real-time applications. Three ablation experiments were conducted for each group, namely type a (as the blank control group), type b (with novel SSA connection blocks added), and type c (with multi-scale feature flows added on top of type b). The results are presented in Table 1. The computation is calculated regarding the number of floating point operators (FLOPs). In all these experiments, we used the loss function defined in Eq. 8.

**Table 1:** Performance comparison, number of parameters, and FLOPs of different models for ablation experiments.

Methods	Type	DICE (%)	JAC (%)	ACC (%)	PRE (%)	SEN (%)	F1-score (%)	Param (M)	FLOPs (G)
ARA-Net	a	10.31	5.43	88.67	40.52	45.00	42.64	16.47	40.29
	b	71.29	55.38	95.13	75.35	68.01	71.49	2.78	30.77
	c	71.63	55.80	95.00	76.25	68.26	72.03	2.78	30.77
ARA-Net (ResNet18)	a	70.40	54.32	94.50	73.60	68.87	71.16	81.53	15.71
	b	73.33	57.90	95.16	73.40	74.32	73.86	108.79	21.07
	c	74.27	59.06	95.17	77.93	72.39	75.06	108.79	21.07
ARA-Net (MobileNet-v3)	a	76.37	61.77	95.51	75.36	78.24	76.77	13.18	3.24
	b	76.15	61.49	95.12	77.66	77.48	77.57	14.35	4.16
	c	77.27	62.96	95.54	83.09	73.94	78.25	14.35	4.16
ARA-Net (EfficientNet-b3)	a	76.64	62.13	95.49	81.50	74.19	77.67	44.51	5.51
	b	77.03	62.65	95.65	82.62	73.18	77.62	57.68	7.17
	c	78.60	64.74	95.82	81.53	77.08	79.24	57.68	7.17

As illustrated in Table 1, among the four experimental groups, the type c model has higher performance metrics in DICE, JAC, and F1-score, yielding a uniform 0.5% improvement compared to type b and a significant improvement compared to the control group, type a. The superior performance achieved by the proposed method with minimal computational overhead increase demonstrates that the novel SSA connection block and MSFF can provide a better solution to the difficulties of segmenting retinal atrophy.

#### 3.3.2. Effectiveness of transfer learning

To measure the effectiveness of using transfer learning, we also set up three groups of experiments, and the results are shown in Table 2. The results show that using transfer learning achieves the best results. Type ‘a’ represents the control groups without transfer learning, and type ‘b’ represents groups that use transfer learning. Note that, in all these experiments, we use the loss function defined in Eq. 8. The application of transfer learning to segmentation methods has been proven to produce significant performance gains.

**Table 2:** Performance comparison of the proposed method with and without using transfer learning.

Methods	Type	DICE (%)	JAC (%)	ACC (%)	PRE (%)	SEN (%)	F1-score (%)
ARA-Net (ResNet18)	a	74.27	59.06	95.17	77.93	72.39	75.06
	b	76.47	61.90	95.75	79.29	75.66	77.43
ARA-Net (MobileNet-v3)	a	77.27	62.96	95.54	83.09	73.94	78.25
	b	82.30	69.91	96.37	86.96	79.17	82.88
ARA-Net (EfficientNet-b3)	a	78.60	64.74	95.82	81.53	77.08	79.24
	b	84.57	72.80	96.95	89.09	80.49	84.57

Specifically, when this strategy was implemented in segmentation tasks, the DICE, JAC, and F1-score realized an average improvement of 4, 6, and 4%, respectively. This demonstrates the potential of transfer learning to develop more accurate segmentation models. Moreover, the boost in performance can result in higher quality predictions of segmentation masks and increased usability in medical applications.

#### 3.3.3. Effectiveness of loss functions

To show the model performance affected by the loss function, we utilize three backbones (ResNet18, MobileNet-v3, and EfficientNet-b3) to build and compare their model performance across three loss functions (BCE loss, Tversky loss, and their combination). The results obtained are shown in Table 3. There is about a 2% improvement of DICE and F1-score when combining the two loss functions compared to using any one of the two loss functions alone.

**Table 3:** Performance comparison of the methods across different loss functions.

Methods	Loss function	DICE (%)	JAC (%)	ACC (%)	PRE (%)	SEN (%)	F1-score (%)
ARA-Net (ResNet18)	BCE	68.74	52.36	94.61	68.97	68.30	68.63
	Tversky Loss	74.91	59.89	94.83	77.28	73.92	75.56
	BCE + Tversky Loss	76.47	61.90	95.75	79.29	75.66	77.43
ARA-Net (MobileNet-v3)	BCE	76.56	62.02	96.18	84.82	69.94	76.66
	Tversky Loss	78.03	63.97	94.81	75.42	84.07	79.51
	BCE + Tversky Loss	82.30	69.91	96.37	86.96	79.17	82.88
ARA-Net (EfficientNet-b3)	BCE	82.39	70.05	96.66	89.43	76.38	82.39
	Tversky Loss	82.99	70.93	96.57	90.37	77.23	83.29
	BCE + Tversky Loss	84.57	72.80	96.95	89.09	80.49	84.57

### 3.4. Comparison study

We compare our proposed method with other UNet-based methods, including UNet++ (Zhou et al., 2018), AttentionUNet (Oktay et al., 2018), R2UNet (Alom et al., 2018), and UNeXt (Valanarasu and Patel, 2022). Table 4 shows the comparison results, demonstrating that our model performs better than the comparison methods. Results from Table 4 indicate that ARA-Net (EfficientNet-b3) performed best among all architectures, achieving a DICE of 84.57%, a JAC of 72.80%, an ACC of 96.95%, a PRE of 89.09%, an SEN of 80.49%, and an F1-score of 84.57%. Further, this was followed by ARA-Net (MobileNet-v3) and UNeXt. However, their performance is commendable due to fewer parameters and computation resources compared to EfficientNet-b3.

**Table 4:** Comparison of segmentation performance, number of parameters, and FLOPs of different methods.

Methods	DICE (%)	JAC (%)	ACC (%)	PRE (%)	SEN (%)	F1-score (%)	Param (M)	FLOPs (G)
UNet++(ResNet-18)	76.84	62.39	96.12	86.67	69.66	77.24	60.92	64.05
AttentionUNet	71.00	55.04	94.50	67.79	78.97	72.95	54.56	67.21
R2UNet	68.13	51.67	94.10	78.29	66.06	71.65	388.88	262.76
UNeXt	77.01	62.62	95.58	80.00	74.71	77.26	5.61	0.57
ARA-Net (ResNet18)	76.47	61.90	95.75	79.29	75.66	77.43	108.79	21.07
ARA-Net (MobileNet-v3)	82.30	69.91	96.37	86.96	79.17	82.88	14.35	4.16
ARA-Net (EfficientNet-b3)	84.57	72.80	96.95	89.09	80.49	84.57	57.68	7.17

**Figure 4:**
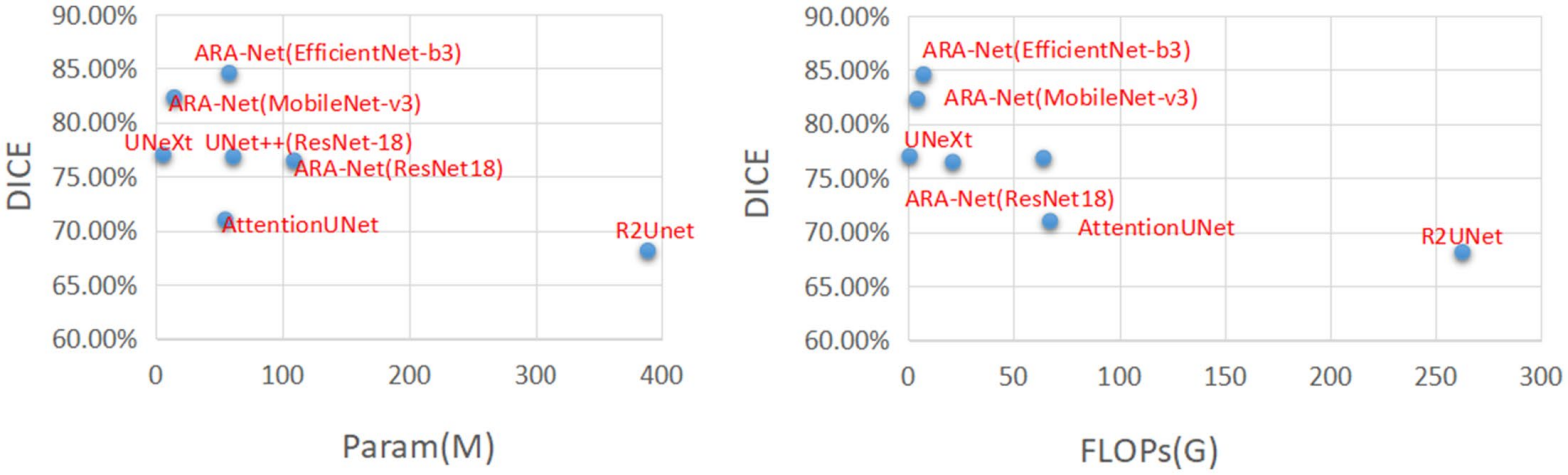
Comparison charts. *X*-axis corresponds to FLOPs(G) and the number of parameters (lower the better). *Y*-axis corresponds to DICE (higher the better).

**Figure 5:**
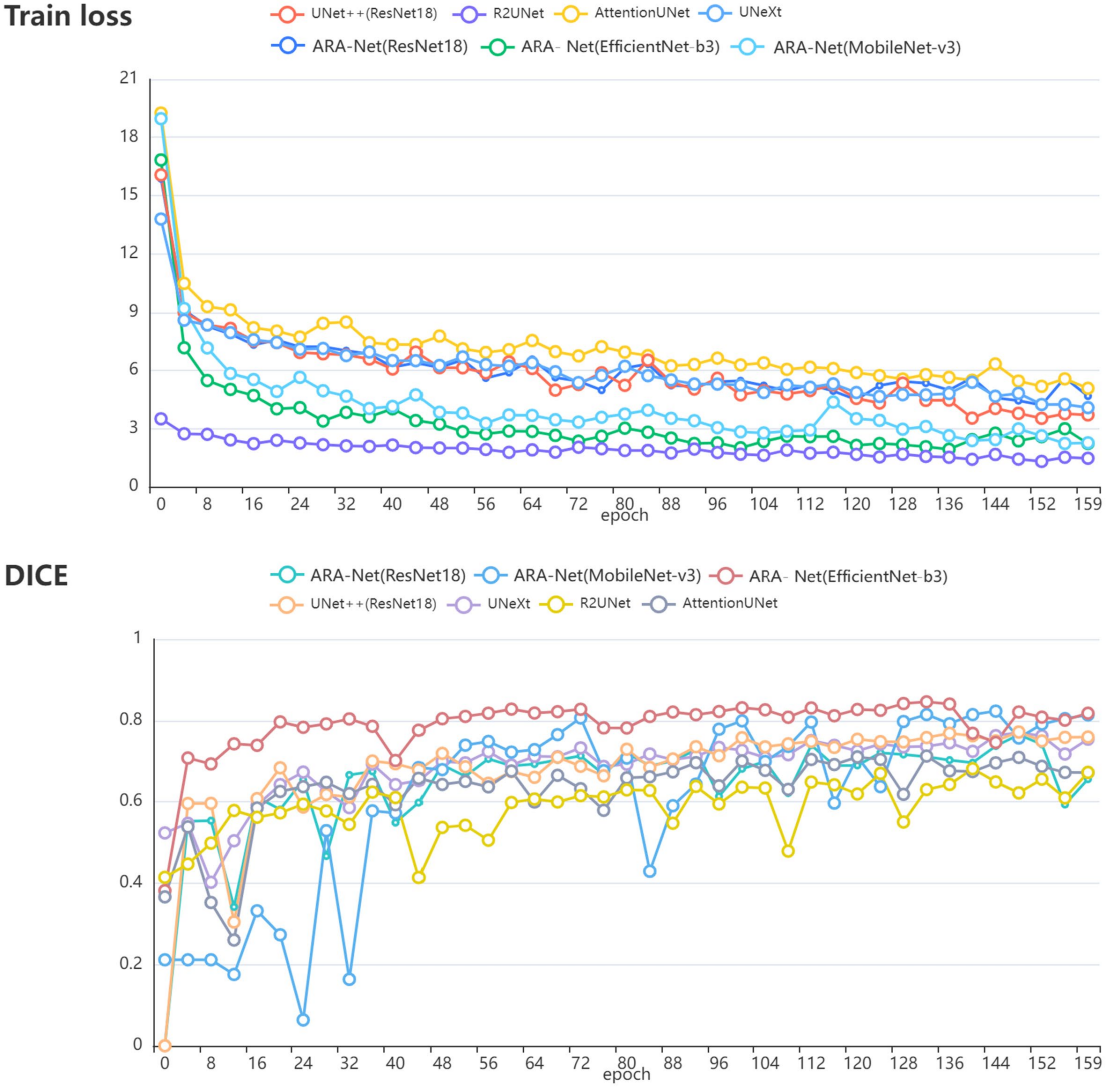
**(A)** The value of the loss function during training. **(B)** The DICE value during validation.

**Figure 6:**
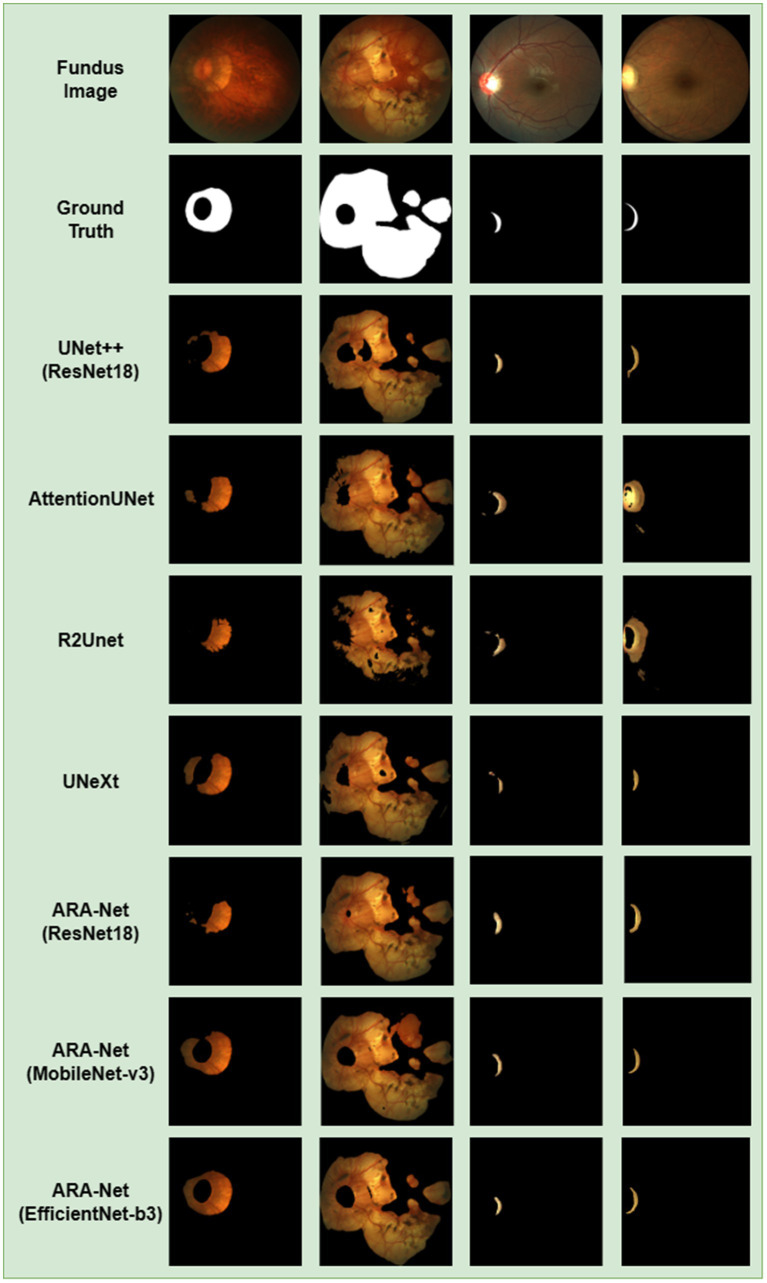
Visual segmentation results for myopic parapapillary atrophy. It shows the color fundus input images, the ground truth masks, and the segmentation results for UNet++ (ResNet18), AttentionUNet, R2UNet, UNeXt, ARA-Net (ResNet18), ARA-Net (MobileNet-v3) and ARA- Net (EfficientNet-b3).

Figure 4 compares DICE scores for various segmentation methods, where the parameters and GLOPs utilized in each method are varied. It can be seen from the results that the ARA-Net (EfficientNet-b3) performs most efficiently in terms of segmentation performance. This is due to the comparatively low GLOPs requirement and the number of parameters needed for this model compared to other methods. UNeXt and ARA-Net (MobileNet-v3) perform significantly better than any other networks in terms of computational complexity and parameter count, which are crucial considerations for practical applications. Figure 5 depicts the change in loss function values during training and DICE values on the validation set. As illustrated in Figure 5, the training process of the networks proceeded gradually, except for R2Unet, which had a more stable loss. On the validation set, the Dice scores for ARA-Net (EfficientNet-b3) and ARA-Net (MobileNet-v3) were both favorable, with ARA-Net (EfficientNet-b3) displaying a more consistent result. Sample qualitative results from various methods are shown in Figure 6. ARA-Net (EfficientNet-b3) and ARA-Net (MobileNet-v3) effectively segment both large and tiny atrophic regions of the retina, as shown in Figure 6. On the other hand, the other networks either produced excessive segmentation of smaller areas or failed to detect more expansive areas of atrophy effectively. Compared to other techniques, ARA-Net (EfficientNet-b3) generates high-quality segmentation predictions, making it a viable option for atrophic area segmentation.

## 4. Discussion

The automated segmentation of the retinal atrophy in fundus images is a valuable tool for ophthalmologists in detecting and diagnosing myopia, as the size of the retinal atrophy is positively correlated with the severity of the condition. Automated segmentation techniques allow for efficient extraction and representation of image features, reducing the need for human intervention.

The advance of deep learning has the potential to further enhance segmentation accuracy through the development of advanced segmentation network models and improved hardware performance. However, the field of automated retinal atrophy segmentation is currently under-researched, with the majority of studies utilizing optical coherence tomography (OCT) images (Fang et al., 2017; He et al., 2021; Szeskin et al., 2021) due to their high image resolution and cross-sectional tissue structure. The limited availability of appropriate datasets, the significant variations in retinal atrophy shape and size among patients, and the interference of blood vessels present significant challenges for applying deep learning approaches to retinal atrophy segmentation. Additionally, the proximity of the retinal atrophy to the optic disc, which can be a source of brightness that can mask the retinal atrophy, further complicates the task. These challenges must be addressed to facilitate progress in retinal atrophy segmentation using deep learning.

The experimental results indicate that other Unet-based models struggle to accurately segment retinal atrophic areas of varying sizes, and the segmentation boundaries are not distinct. To address this challenge, novel SSA connection blocks and multi-scale feature flows were incorporated into the model to extract boundary information and shape features of the atrophic regions. The SSA block includes the initial skip connection as well as the PPSA branch. The polarized filtration and enhancement are two crucial modules. By reducing the dimensions of the inputting data while maintaining the accuracy of the channel and spatial attention, our model can focus on the most critical features and retaining critical details, leading to better feature extraction and more accurate predictions. In addition, the enhancement strategy maps the model’s results to a desired distribution, resulting in more accurate predictions.

The proposed model demonstrated superior segmentation results on atrophic regions of different sizes, as shown in Figure 6. Additionally, during the training phase, the ARA-Net model utilized three pre-training models (ResNet, MobileNet-v3, and EfficientNet-b3) to extract richer feature information, resulting in improved performance. This approach leverages pre-trained models on large datasets to initialize the model weights, allowing the model to adapt to new datasets with limited training data quickly. Furthermore, using a combination of BCE loss and TVERSKY loss during training enabled the model to focus more on small ROIs, reducing the likelihood of predicting small-size retinal atrophy areas as background.

Due to the scarcity of data, the proposed method can achieve better results to some extent depending on the pre-trained model of ImageNet. In the training phase, we use pre-trained model of ImageNet for training the ARA-Net. As we known, the pre-trained model of ImageNet is trained on the images collected from natural environment, and the model is not specific to the medical domain. Thus, we might use some pre-trained model based on medical images to substitute the pre-trained model of ImageNet, and investigate the effect of different image domain based pre-trained model on the segmentation performance for retinal atrophy areas. Meanwhile, future study might consider utilizing other data improvement methods, such as the use of generative models for data augmentation or the integration of multimodal imaging. Additionally, further improvements can consider few-shot learning methods and design segmentation networks for different myopic stages of retinal atrophy. As retinal atrophy can vary in size and shape across different stages, it is crucial to develop segmentation techniques tailored to each stage. These enhancements hold the potential to improve the accuracy of retinal atrophy segmentation, leading to more precise diagnosis and treatment.

## 5. Conclusion

In this work, we proposed an ARA-Net model for segmenting the retinal atrophic area from 2D fundus images. In particular, our proposed novel skip-connect blocks named PPSA effectively fuse the feature maps between the encoder and decoder, enabling the network to learn representational feature maps from both channel and spatial dimensions. The MSFF utilized by our model also help to enhance the semantic information of the images, and the combined Tversky and BCE loss functions further improve the model performance. Comprehensive experimental results demonstrate that our proposed ARA-Net model achieves awesome performance on retinal atrophy segmentation, especially in challenging scenarios with blurred boundaries and irregular shapes. Our work has made significant contributions in solving the retinal atrophy segmentation challenges and introducing the PPSA blocks as a new technique for feature fusion in medical image segmentation. Comprehensive experimental results demonstrate the effectiveness of ARA-Net and its potential clinical value in retinal atrophic area segmentation applications.

## Data availability statement

The original contributions presented in the study are included in the article/Supplementary material, further inquiries can be directed to the corresponding author.

## Author contributions

LC and ML contributed to the conception of the study. LC performed the experiments and wrote the first draft of the manuscript. YZ, SG, and ML performed the data analysis and revised the manuscript. ZW and HT revised the manuscript and supervised the entire study. All authors contributed to the article and approved the submitted version.

## Conflict of interest

The authors declare that the research was conducted in the absence of any commercial or financial relationships that could be construed as a potential conflict of interest.

## Publisher’s note

All claims expressed in this article are solely those of the authors and do not necessarily represent those of their affiliated organizations, or those of the publisher, the editors and the reviewers. Any product that may be evaluated in this article, or claim that may be made by its manufacturer, is not guaranteed or endorsed by the publisher.
